# A dual nature of γδ T cell immune memory responses

**DOI:** 10.7554/eLife.104887

**Published:** 2025-07-23

**Authors:** Tsz Kin Suen, Burcu Al, Alice Scarpa, Anca Dorhoi, Mihai G Netea, Katarzyna Placek

**Affiliations:** 1 Immunology and Metabolism Unit, Life and Medical Sciences (LIMES) Institute, University of Bonn Bonn Germany; 2 Institut of Immunology, Friedrich-Loeffler-Institut, Federal Research Institute for Animal Health Greifswald-Insel Riems Germany; 3 Department of Internal Medicine and Radboud Center for Infectious Diseases, Radboud University Medical Center Nijmegen Netherlands; Queen's University Canada; Indian Institute of Science Education and Research (IISER) India

**Keywords:** immune memory, gamma delta T cells, trained immunity

## Abstract

Immune memory was considered for decades an exclusive hallmark of the adaptive immune response. However, recent studies have revealed that innate immune cells can also ‘recall’ information of a primary insult during infection or vaccination and deploy robust antigen-agonistic immune reactivity upon secondary challenge. This *de-facto* innate immune memory response is designated as ‘trained immunity’. γδ T cells are unconventional T cells that possess unique immunologic features of both adaptive and innate immunity. Their immune memory responses to various bacterial and viral agents were originally described to be of an adaptive immune nature. Nevertheless, growing evidence shows that γδ T cells can also mount antigen-independent memory responses resembling trained immunity. In this review, we discuss the dual nature of immune memory responses of γδ T cells and provide insights into their important role in protection against bacterial, viral, and parasitic infections in humans and animals.

## Introduction

Multicellular organisms are continuously challenged with potentially deadly infections caused by various microbial pathogens. To counteract these dangerous encounters, they acquired the capacity to detect microbial agents and activate suitable defense mechanisms, constituting the immune response. Traditionally, the immune response has been categorized into innate and adaptive arms with underlying cellular components. However, considering recent progress in understanding the complexity of the immune system, this distinction is becoming increasingly challenging to define. First, because some of the immune cell populations, such as innate lymphoid cells or gamma delta (γδ) T cells exhibit properties of both adaptive and innate immunity (Eberl et al., 1979; Vivier et al., 2008; Vantourout and Hayday, 2013). Second, because immune memory is no longer seen as a characteristic unique to adaptive immunity, but innate immune cells have also been recently shown to have the capacity to store and recall information of previous stimulations (Netea et al., 2020). Paradigm changes raise new questions and one of these relates to unconventional γδ T cells: does their memory have adaptive or innate features or both? In this review, we discuss recent findings on the memory responses of γδ T cells in a species-wide context, considering the evolution-driven variabilities of this cell population. We emphasize the relevance of memory features for the host defense against various infectious agents and frame outstanding questions to advance our understanding about the immunobiology of γδ T cells. While acknowledging the critical roles of γδ T cells in sensing normality, the role of their memory patterns in cancer is beyond the scope of this review, as their potential for immunotherapies has been excellently discussed recently (Hayday et al., 2024).

## Innate vs. adaptive immune responses

Innate immunity emerged as the first protective antimicrobial strategy during the evolution of multicellular life (Janeway, 2001). Phagocytes such as monocytes, macrophages, granulocytes, and dendritic cells, which constitute the innate immune system, are first to react to an infection. They sense and respond to pathogens thanks to germline-encoded receptors termed pattern recognition receptors (PRRs), such as Toll-like receptors (TLRs), C-type lectin receptors (CLR), Nod-like receptors (NLRs), cyclic GMP-AMP synthase (cGAS)-like receptors (cGLR), retinoic acid-inducible gene I (RIG-I)-like receptors (RLRs), and scavenger receptors (SR), which recognize evolutionarily conserved microbial structures called pathogen-associated molecular patterns (PAMPs). Depending on the PRR-PAMP interaction, distinct signaling pathways and transcriptional programs are initiated, leading to different antimicrobial responses (Akira et al., 2006). Commonly, phagocytes engulf the microbe and directly destroy it, subsequently producing chemokines and cytokines to propagate the immune reaction (Fu and Harrison, 2021). While innate immune cells are effective in eliminating microbes, in some cases, the more potent and specific adaptive immune response needs to be elicited. To achieve that, activated innate immune cells produce cytokines and present antigens from phagocytosed microbes to initiate adaptive immune responses. Adaptive immune cells, such as B cells and T cells, which are activated later in the course of infection and in an antigen-specific manner, generate a targeted, diversified, and robust immune reaction that consists of antibody release by B cells, cytotoxicity towards infected cells, and cytokine production by T cells. The specificity of adaptive immune reaction towards an antigen is conferred in the expression of highly antigen-specific receptors: B cell receptor (BCR) and T cell receptor (TCR) on the surface of B and T cells, respectively. Highly diverse BCRs and TCRs are generated during adaptive immune cell development as a result of somatic recombination of the BCR and TCR loci (Bassing et al., 2002). These receptors are, therefore, not inherited, in contrast to PRRs, but are acquired and propagated due to the clonal expansion of T cells and B cells bearing antigen-specific receptors during the lifetime of an organism, making the adaptive immune system highly specific and personalized. Furthermore, the effectiveness of the defense mechanisms in conferring survival of an organism in a given environment is amplified by the immune memory responses, which enable faster and more effective immune reactions upon a secondary challenge. For decades, memory features have been solely attributed to adaptive immunity, whose long-lived components, memory T and B cells, and specific antibodies are primary sources of adaptive immune memory. Upon the resolution of the infection, they persist in circulation, bone marrow, or at tissue sites, ready to react rapidly when rechallenged with the same antigen. Memory B and T cells are able to respond faster and more effectively compared to the naïve cells due to epigenetic reprogramming, which involves the redistribution of suppressive DNA methylation on gene loci related to immune activation (Mittelstaedt et al., 2021). Furthermore, the gain of activating histone modifications, e.g., acetylation of histone (H) 3 or methylation of lysine (K) 4 on H3 on cytokines, cytotoxic molecules and other immune activation-related loci during the transition from naïve to memory T cells allows rapid target gene activation upon restimulation (Henning et al., 2018; Araki et al., 2008; Barski et al., 2017). The redistribution of chromatin modifications that persist upon the removal of the initial TCR stimulus results in a poised chromatin state at effector genes in memory T cells. This poised chromatin environment does not license gene transcription but enables a rapid reactivation of effector genes upon secondary stimulation (Denton et al., 2011; Fann et al., 2006; Araki et al., 2008; Barski et al., 2017; Zediak et al., 2011). The accelerated response to antigens by memory T cells, which rapidly proliferate and abundantly produce effector molecules, is also sustained by metabolic reprogramming (Buck et al., 2015). In this regard, memory T cells have increased mitochondrial mass and spare respiratory capacity compared with naïve T cells, implementing higher readiness of metabolic programs to accommodate the increased energy requirements of memory T cells upon antigenic stimulation.

**Figure 1.**
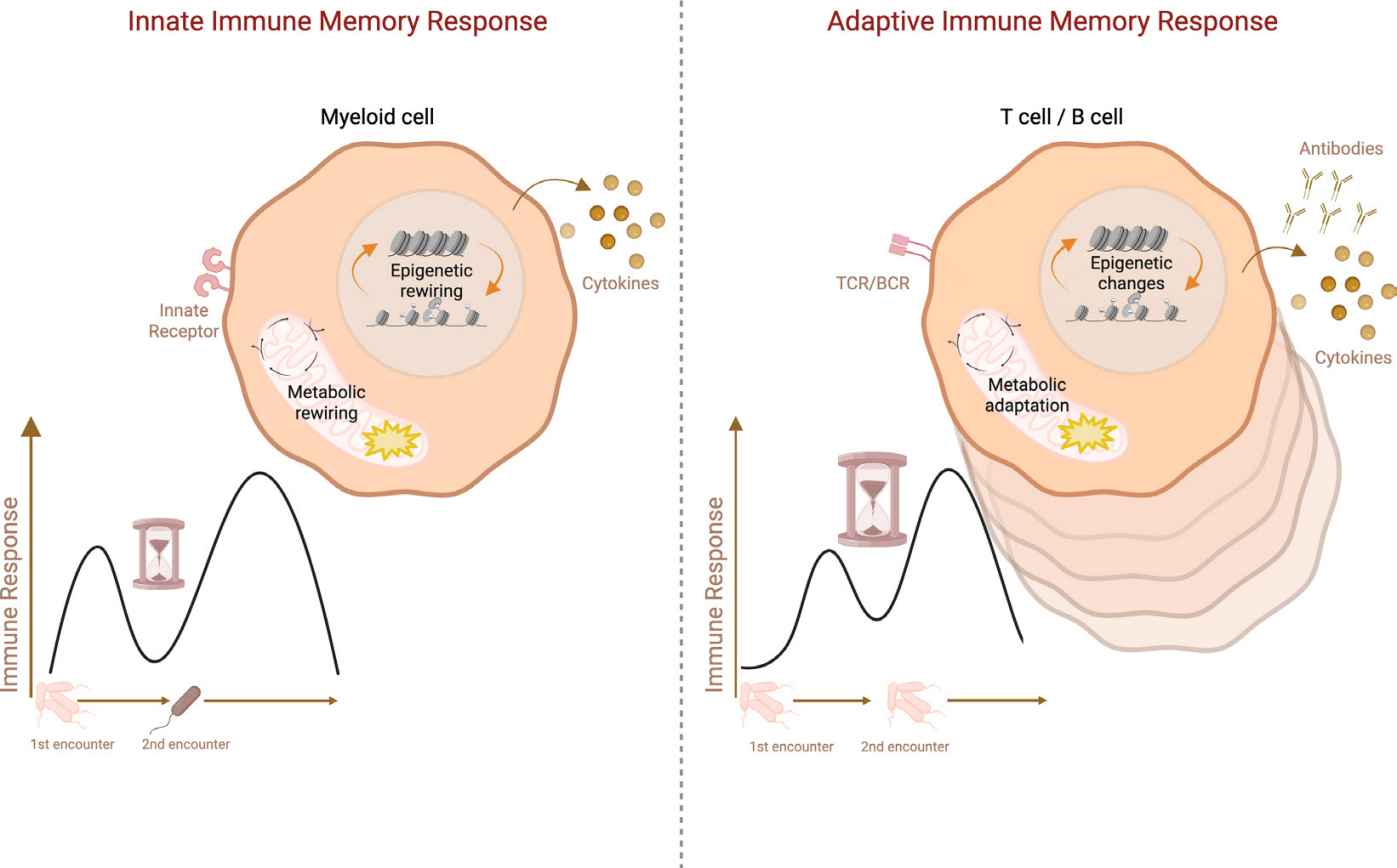
Schematic representation of adaptive vs. innate immune memory characteristics. Myeloid cells and lymphocytes mount immune memory responses characterized by the enhanced effector function upon secondary exposure. While innate immune cells produce more inflammatory cytokines upon secondary challenge with heterologous stimuli, adaptive memory immune cells rapidly proliferate and generate copious amounts of antibodies and cytokines upon rechallenge with the same antigen. Antibodies, as well as memory B cells and T cells, persist in the host while cytokines produced by innate immune cells return to the baseline after the resolution of infection. Innate immune memory lasts relatively shorter than adaptive immune memory. Both adaptive and innate immune memory formation is accompanied by epigenetics and metabolic rewiring, facilitating transcriptional responses and allowing more robust immune reactions upon secondary challenge. TCR: T-cell receptor, BCR: B-cell receptor. Created with BioRender.com.

Increasing evidence points to the generation of immune memory responses also by innate immune cells. In contrast to adaptive memory responses, innate immune memory, also called ‘trained immunity’ (Netea et al., 2020), is not specific to the initial insult, e.g., epitope or antigen (Figure 1). Innate immune memory was originally discovered in monocytes and macrophages in response to *Candida albicans* infection and its wall component β-glucan, as well as upon vaccination with *Mycobacterium bovis* Bacille Calmette-Guerin (BCG) (Netea et al., 2011). Similar to memory B cells and T cells, trained innate immune cells undergo metabolic and epigenetic reprogramming in order to sustain their more robust response upon rechallenge. They exhibit augmented glycolysis, oxidative phosphorylation (OXPHOS), activation of metabolic pathways such as cholesterol synthesis and glutaminolysis, as well as accumulation of Krebs cycle metabolites including succinate and fumarate (Cheng et al., 2014). This metabolic reprogramming is closely associated with epigenetic rewiring (Fanucchi et al., 2021). Immune response genes accumulate activating H3K4 trimethylation at promoters and H3K4 monomethylation and H3K27 acetylation at enhancers, which afford more robust gene expression upon reactivation in trained immune cells (Quintin et al., 2012; Novakovic et al., 2016; Saeed et al., 2014). Similar to poised chromatin in memory T cells (Denton et al., 2011; Fann et al., 2006; Araki et al., 2008; Barski et al., 2017; Zediak et al., 2011), latent enhancers confer memory of environmental exposure in macrophages (Ostuni et al., 2013). Contrary to adaptive immune memory, the induction of trained immunity also takes place at the level of myeloid progenitors in the bone marrow (Mitroulis et al., 2018). Similar to innate immune cells in the periphery, myeloid progenitors undergo epigenetic and metabolic changes which facilitate enhanced innate immune cell responses (Mitroulis et al., 2018; Cirovic et al., 2020).

The ability of innate immune cells to adapt their functional programs based on previous encounters with microbes and produce a stronger response following subsequent infections is hypothesized to underlie heterologous protection induced by live attenuated vaccines, including BCG, oral polio vaccine (OPV), and measles-mumps-rubella (MMR) (Benn et al., 2013). While trained immunity can enhance host defense against infections, it can also backfire, potentially leading to maladaptive responses and inflammatory diseases. This maladaptation has been linked to various inflammatory conditions such as gout, rheumatoid arthritis, periodontitis, infections, and atherosclerosis (Flores-Gomez et al., 2021; Badii et al., 2022; Cabău et al., 2020; Li et al., 2023c). It is, therefore, crucial to determine in which context boosting innate immune memory is beneficial and when it turns detrimental for the host. It is also important to scrutinize the cellular components involved in trained immunity to enable the design of better prevention and treatment strategies for many diseases. γδ T cells emerge as the most recently described immune cell type endowed with trained immunity potential.

## The subtle art of being unconventional: γδ T cells

γδ T cells are a distinct subset of T cells with unique attributes. The primary functions of these cells are to provide rapid responses to preserve tissue integrity, maintain immune and tissue homeostasis, detect and combat cancer, regulate nutrient uptake (Sullivan et al., 1979), and support barrier functions against invaders (Hayday, 2000; Wu et al., 2014). Despite being discovered four decades ago (Hayday et al., 1985; Saito et al., 1984), γδ T cells remain largely enigmatic compared to well-studied alpha beta (αβ) T cell counterparts. The current knowledge stems primarily from the analysis of this population in humans and murine models. As their name indicates, γδ T cell receptor (TCR) consists of one gamma and one delta chain, which exhibits different modes of antigen recognition than αβ TCRs (Ribot et al., 2021). While αβ TCRs recognize peptide antigens presented by antigen-presenting cells (APCs) in the context of major histocompatibility complex (MHC) molecules, the ligands for γδ TCRs, although still largely unknown, are nonpeptidic and can be of exogenous and endogenous origin and do not depend on MHC presentation. Interestingly, a recent study suggests that some γδ TCRs are polyspecific and, in contrast to highly antigen-specific αβ TCRs, can recognize multiple structurally diverse molecules (Guo et al., 2024). CD4 and CD8 TCR coreceptors are rarely expressed on γδ T cells, unlike on αβ T cells, reflecting the MHC-independent activation. CD8 expression is restricted to a subset of γδ T cells that are mainly found at mucosal sites and in chronic infection or inflammatory conditions (Hayday et al., 2001; Holderness et al., 2013; Gaballa et al., 2019b; Kadivar et al., 2016; Gaballa et al., 2019a). Aside from TCR, γδ T cells express innate immune receptors such as natural killer group 2 member C (NKG2C) and NKG2D (Rincon-Orozco et al., 2005; Fausther-Bovendo et al., 2008). These receptors recognize stress molecules and, upon stimulation, induce cytokine and cytotoxic granule production, such as perforin and granzymes (Sandoz et al., 2023). Furthermore, γδ T cells can express TLRs, such as TLR2, TLR3, and TLR6 (Pietschmann et al., 2009) and nucleotide-binding oligomerization domain containing 2 (NOD2) receptor (Marischen et al., 2011; Kerns et al., 2009). TLR and NOD2 ligands co-stimulate TCR-activated γδ T cells, leading to enhanced cytokine and chemokine production (Pietschmann et al., 2009; Wesch et al., 2006; Deetz et al., 2006). Recently, a co-stimulatory effect of Stimulator of Interferon Genes (STING) receptor ligands in γδ T cells has also been reported (Serrano et al., 2022).

Similar to αβ TCR, the assembly of γ and δ chains requires recombination of variable (V), diversity (D), and joining (J) gene fragments during thymic development, which drives antigen receptor diversity. This diversity is excessively increased by random gain and loss of nucleotides at the junction sites between the various segments (Willcox et al., 2018). This untemplated process enables the generation of flexible and exceptionally long complementarity-determining region 3 (CDR3) loops, the most variable one compared to CDR1 and CDR2 (Rock et al., 1994; Xin et al., 2024). CDRs are polypeptide sequences within TCRs that play crucial roles in dictating antigen recognition and binding. Especially, CDR3 in the d chain tends to be longer and more flexible than its αβ counterpart (Pellicci et al., 2014). These stem-like extended loops provide the TCR a physical ability to reach and mould around unconventional ligands with different sizes and shapes. Therefore, γδ T cells can overcome ancestral sequence-specific restrictions and lower receptor diversity with this enhanced conformational plasticity, allowing them to recognize a broad range of pathogens and non-peptide ligands (Chen et al., 2008; Legut et al., 2015). Bypassing sequence-specific limitations further contributes to their innate-like memory and rapid recall response features. Taken together, the diversity potential of γδ TCRs is greater than that of αβ T cells and B cells, yet it is not fully realized (Carding and Egan, 2002; Hu et al., 2023). The rearrangement of the *TRG* locus encoding γ chain precedes the rearrangement of the *TRB* locus and is followed by the DNA recombination at the *TRD* locus. Upon successful VDJ rearrangement of both γ and δ chains, cells commit to the γδ T cell lineage and undergo a ‘γδ selection’ process in which the γδ TCR signaling confirms the functionality of the receptor (Hayes et al., 2005; Muro et al., 2018; Prinz et al., 2006; Taghon et al., 2006). It is not well established whether γδ TCR signaling during thymic development is ligand-dependent (Zeng et al., 2012; Muro et al., 2019). Based on TCR chain composition, γδ T cells are classified into distinct subsets. In general, delta chain-based and gamma chain-based classifications are used in primates and mice, respectively.

### *Primate* γδ *T cells*

The main human γδ T cell subsets are Vδ1, Vδ2, Vδ3, and Vδ5 (nomenclature by Lefranc and Rabbitts LeFranc et al., 1986; Wu et al., 2017). Of them, the best characterized are Vδ2 T cells. The Vδ2 chain predominantly pairs with γ9 chain (LeFranc et al., 1986; Davey et al., 2018; Hinz et al., 1997) and these Vγ9^+^Vδ2^+^ T cells consist of up to 90% of the whole γδ T cell population in human peripheral blood (Wu et al., 2014). Although most studies do not discriminate between Vγ9^+^ and Vγ9^-^ Vδ2 T cell subpopulations due to the scarcity of the latter, recent studies suggest that these cell subpopulations have distinct features: Vγ9^+^Vδ2^+^ bearing characteristics of innate immunity and Vγ9^-^Vδ2^+^ having adaptive immune character (Davey et al., 2018). The antigens that activate Vγ9^+^Vδ2^+^ T cells comprise phosphoesters, alkylamines, nucleotide conjugates and heat shock proteins that are expressed by microbial pathogens but also by host cells (Fisch et al., 1979; Haregewoin et al., 1989; Bukowski et al., 1999; Constant et al., 1994; Tanaka et al., 1995). In particular, the Vγ9^+^Vδ2^+^ TCR recognizes phosphoantigens such as (E)–4-hydroxy-3-methyl-but-2-enyl pyrophosphate (HMB-PP) and isopentenyl pyrophosphate (IPP), the intermediate metabolites of the 2-C-methyl-D-erythritol 4-phosphate (MEP) pathway in many bacteria and the mevalonate pathway in mammalian cells, respectively (Tanaka et al., 1995; Gu et al., 2014; Simões et al., 2018; Tyler et al., 2015; Hintz et al., 2001). The phosphoantigens are identified by Vδ2 T cells in the context of Butyrophilin 2A1 and 3A1 molecules (Laplagne et al., 2021; Cano et al., 2021; Fulford et al., 2024). Numerous in vivo and in vitro studies point to the involvement of Vδ2 T cells in the defense against malignant cells (Schönefeldt et al., 2021) and various pathogens such as *M. tuberculosis* (Dieli et al., 2000; Chen, 2016), *Plasmodium falciparum* (Hernández-Castañeda et al., 2020; Howard et al., 2018; Junqueira et al., 2021), *Listeria monocytogenes* (Ryan-Payseur et al., 2012), *Brucella suis* (Bessoles et al., 2011; Oliaro et al., 2005), human immunodeficiency virus (HIV) (He et al., 2013; Poccia et al., 1999), influenza virus (Qin et al., 2011; Qin et al., 2009), hepatitis C virus (Cimini et al., 2018), severe acute respiratory syndrome coronavirus SARS-CoV (Poccia et al., 2006), and possibly many others (Gay et al., 2022). The effector functions of Vδ2 T cells encompass a wide range of activities. Upon activation, Vδ2 T cells produce mainly TNF and IFN-γ, but are also able to secrete IL-17 (Ness-Schwickerath et al., 2010) or IL-4 (Wesch et al., 2001) in certain conditions. Furthermore, they directly kill target cells by cytotoxic activity (Dieli et al., 2000; Tokuyama et al., 2008), phagocytosis (Junqueira et al., 2021), induction of apoptosis via Fas-Fas ligand interactions (Li et al., 2011), antibody-dependent cellular cytotoxicity (ADCC) (Gertner-Dardenne et al., 2009; Capietto et al., 2011; Tokuyama et al., 2008) or by mobilizing other immune cells (Hu et al., 2023). For example, professional antigen-presenting capabilities (Junqueira et al., 2021; Brandes et al., 2005) and B cell helper function (Caccamo et al., 2006) were also described in Vδ2 T cells. The lack of pathogen specificity and the huge functional plasticity of Vδ2 T cells make them a unique player in an immune reaction. Vγ9^+^Vδ2^+^ T cells have also been described in non-human primates (Kazen and Adams, 2011; Wang et al., 2003) and alpacas (Fichtner et al., 2020b) but no such cell population exists in mice.

The second most abundant γδ T cell population in the human peripheral blood are Vδ1 T cells, which are enriched at barrier tissues (Qu et al., 2022). The Vδ1 chain is known to pair with various γ chains, including γ2, γ3, γ4, γ5, and γ8. The cells are largely found in the skin, gut, liver, spleen, lung, and bone marrow and recognize CD1 lipid-presenting molecules via their TCR (Spada et al., 2000) as well as stress-inducible MICA and MICB molecules upon tumor transformation or viral infection (Groh et al., 1998; Groh et al., 1999), although this recognition is likely not mediated by the TCR (Bauer et al., 1999). Functionally, they resemble cytotoxic, Th1-like phenotypes characterized by IFN-γ expression (Deusch et al., 1991). Apart from killing various epithelial tumors (Maeurer et al., 1996), Vδ1 T cells are also involved in tissue homeostasis and wound healing by producing insulin-like growth factor-1 (Toulon et al., 2009). Cytomegalovirus (CMV) and possibly HIV and *P. falciparum* drive TCR-dependent expansion of Vδ1 T cells (Hviid et al., 2001; De Maria et al., 1992; Vermijlen et al., 2010; Déchanet et al., 1999; McMurray et al., 2022; Ho et al., 1994; Worku et al., 2003; von Borstel et al., 2021), yet the role of Vδ1 T cells in the defense against these pathogens is not clear. Based on clonal expansion of Vδ1 T cells observed upon viral and parasitic infections and consequent generation of a long-lived, TCR-focused effector T cell population, they are considered to have an adaptive immune character (von Borstel et al., 2021; Davey et al., 2017; Ravens et al., 2017; McMurray et al., 2022; Hunter et al., 2018; Rutishauser et al., 2020). Yet, liver-resident Vδ2^-^ T cells, including Vδ1 T cells, have been shown to be polyfunctional and responsive to both TCR and innate stimuli (Hunter et al., 2018). Vδ3 T cells are a minor human γδ T cell population in peripheral blood but are mainly located in the liver, gut, bone marrow, and lymph nodes and significantly expand in certain pathological conditions (Hunter et al., 2018; Dunne et al., 2013; Kenna et al., 2004; Kabelitz et al., 1997; Falk et al., 2008). They recognize the stress-related molecules CD1d and annexin-A2 (ANX) (Mangan et al., 2013; Marlin et al., 2017) and produce mainly TNF (Petrasca et al., 2018). Although there are limited reports on the function of Vδ3 T cells, they have been shown to induce dendritic cell (DC) maturation to cytokine-producing APCs (Mangan et al., 2013) and B cell maturation to IgM-secreting cells (Petrasca et al., 2018). Vδ5 T cells are a little-known subset recognizing stressed cells via the endothelial protein C receptor (EPRC), yet their functions remain largely elusive (Willcox et al., 2012). Recent findings indicate that a more diverse δ chain repertoire exists, particularly in peripheral blood and liver, including Vδ4, Vδ6, Vδ7, and Vδ8 (Hunter et al., 2018; Zheng et al., 2014; Christopoulos et al., 2016; Wang et al., 2014). Yet, these cells were only detected in pathological conditions so far, and there is still a lack of knowledge about their properties, such as gamma chain pairing and effector functions.

### Mouse γδ T cells

γδ T cell subpopulations are not very well conserved between humans and mice. Vγ1, Vγ2, Vγ3, Vγ4, Vγ5, Vγ6, and Vγ7 subsets have been identified in mice so far (Heilig and Tonegawa’s nomenclature [Heilig and Tonegawa, 1986]) adopted in this review (McMurray et al., 2022). The γ chains predominantly found in mouse γδ TCRs are Vγ1 and Vγ4, which pair with a variety of delta chains (He et al., 2010). Vγ1 and Vγ4 T cells are found in peripheral blood, secondary lymphoid organs, liver, and lung (He et al., 2010; Pereira et al., 1995; Qi et al., 2021). Resident Vγ1 T cells in lymphoid tissues support B cell differentiation and antibody production upon vaccination or infection (Ullrich et al., 2021; Huang et al., 2009), while in the lung, they promote allergic airway hyperresponsiveness (AHR) by synergizing with invariant natural killer T (iNKT) cells (Jin et al., 2009; Hahn et al., 2004). They have also been shown to protect from Coxsackievirus B3 infection-induced myocarditis by promoting Th2 responses (Huber et al., 2000). Vγ4 T cells present in the dermis primarily produce IL-17A during mycobacterial infection to further promote neutrophil infiltration to the skin (Sumaria et al., 2011). Mucosal and hepatic Vγ4 T cells play an important role in protecting the mice from *L. monocytogenes* infection by producing IFN-γ and IL17A and collaborating with αβ T cells (Sheridan et al., 2013; Hamada et al., 2008b; Khairallah et al., 2022). In the lung, this subset might play an anti-inflammatory or pro-inflammatory role depending on invading pathogens and environmental signals, as shown in AHR (Hahn et al., 2003) or Coxsackievirus B3 infection (Huber and Sartini, 2005). It is also interesting to note that Vγ1 and Vγ4 T cells are shown to have opposing roles in selected disease models (Hahn et al., 2004; Huber et al., 2000). For example, as mentioned, AHR is promoted by Vγ1 cells but suppressed by Vγ4 cells in a mouse asthma model (Hahn et al., 2004), while the susceptibility to Coxsackievirus B3-induced myocarditis is suppressed by Vγ1 cells but promoted by Vγ4 cells (Huber et al., 2000).

Apart from Vγ1 and Vγ4, Vγ6 T cells are also present in the mouse lung (Sim et al., 1994; Hayes et al., 1996). Upon infection, all these three subsets proliferate and secrete distinct chemokines and cytokines, such as CXCL2 and TNF, to recruit other immune cells, mainly neutrophils (Nakasone et al., 2007). The localization of Vγ6 T cells is not limited to the lung; they are broadly distributed in various tissues, including the peritoneal cavity (O’Brien et al., 2010), reproductive tract (Itohara et al., 1990), tongue (Itohara et al., 1990), placenta/decidua (Heyborne et al., 1992; Pinget et al., 2016), dermis (Cai et al., 2014; Tan et al., 2019), gingiva (Wilharm et al., 2019), nasal epithelium (Kim et al., 2008) and tendon-to-bone attachment sites (Reinhardt et al., 2016). These cells have been found to expand in response to a variety of bacteria (e.g., *Listeria* [Sheridan et al., 2013; Hamada et al., 2008a; Ikebe et al., 2001], *Escherichia coli* [Shibata et al., 2007], *Bacillus subtilis* [Simonian et al., 2006], *M. tuberculosis* [Umemura et al., 2016], *Streptococcus pneumoniae* [Paget et al., 2015], and *Staphylococcus aureus* [Hamada et al., 2008b; Marchitto et al., 2019]) and in several disease models, including pulmonary fibrosis (Simonian et al., 2009), nephritis (Wu et al., 2004), and testicular inflammation (Mukasa et al., 1999). Vγ5 T cells are the predominant population in the epidermis of rodent skin, where they maintain their homeostatic density throughout life by self-renewal capacity. They are also called dendritic epidermal T cells (DETC) and have unique roles in wound healing and immune surveillance (Sutoh et al., 2018). In stressed skin, DETCs are activated, resulting in elevated IFN-γ and IL-17 production as well as cytotoxic properties (Nielsen et al., 2015). Lastly, the Vγ7 subset is most abundant in the epithelial layer of the gut, playing essential roles in maintaining intestinal homeostasis and responding to pathogens or tissue damage by producing mainly IFN-γ together with a wide range of other cytokines such as TNF, TGF-β, IL-10, IL-13, and displaying high cytotoxic potential (Li et al., 2023b; Di Marco Barros et al., 2016). Interestingly, it has been shown that these cells play different roles in the different stages of colitis: a pathogenic role in the early stage but a protective role in the later stage (Kühl et al., 2007). There is also a significant population of Vγ6 T cells in the gut lamina propria that predominantly produces IL-17 and IL-22 to limit bacterial invasion and intestinal inflammation (Rampoldi and Prinz, 2022).

### γδ T cells in other species

γδ T cells are found in all jawed vertebrates (Holderness et al., 2013). Although best described in humans and mice, different subsets of γδ T cells have been increasingly characterized in cattle, swine, sheep, chickens, fish, and reptiles (Holderness et al., 2013). Artiodactyls, specifically cattle and swine, have relatively high frequencies of γδ T cells in blood, accounting for up to 60% of circulating lymphocytes, especially in young animals (Talker et al., 2013; Guzman et al., 2014). The classification of γδ T cell subsets is also different in these species, mainly based on the expression profiles of scavenger receptor family workshop cluster 1 (WC1), CD2, and CD8, as it has been reported for pigs (Le Page et al., 2021). Cattle γδ T cells are identified as WC1^−^, WC1.1^+^, and a WC1.2^+^ subsets and are involved in responses to severe infections such as that caused by foot-and-mouth disease virus (FMDV) (Wilharm et al., 2019; Toka et al., 2011). They display a great proliferative response in the peripheral blood of animals infected with bovine herpesvirus type I and bovine diarrhea virus infections (Silflow et al., 2005; Amadori et al., 1995). While WC1.2^+^ γδ T cell clones in cattle proliferate and secrete IFN-γ in response to *Anaplasma marginale* (Lahmers et al., 2006; Lahmers et al., 2005), *M. tuberculosis* elicits preferential activation of the WC1.1^+^γδ T cell subset (Bhat et al., 2023). Although γδ T cells strongly proliferate upon mycobacterial stimulation ex vivo, they release minute amounts of IFN-γ (Smyth et al., 2001). In swine, γδ T cells represent an important source of IFN-γ and IL-17 production, express cytotoxic activity and orchestrate both innate and adaptive immune responses upon infection with reproductive and respiratory syndrome virus infection (Le Page et al., 2022; Olin et al., 2005). Deletion of γδ T cells does not alter the structure of immune organs or the health of conventionally housed pigs but may reduce responses to live-attenuated vaccines (Petersen et al., 2021). Birds, such as chickens, have a high frequency of γδ T cells in the intestinal mucosa. During infection with Eimeria acervulina, an early increase of local intestinal γδ T cells has been observed, reflecting their role in early host defense (Choi and Lillehoj, 2000). While the absence of γδ T leaves chicken health under conventional breeding unaltered (von Heyl et al., 2023), it does cause susceptibility to an avian oncogenic alphaherpesvirus (Sabsabi et al., 2024). There is further evidence that γδ T cells are present in other species, yet their exact subsets and mechanisms of action are not well characterized to date. Interestingly, certain vertebrates, such as squamate reptiles, are unique in that they lost γδ T cells (Morrissey et al., 2022). The lack of TCRγ and TCRδ transcripts seems to be due to large genome deletions in the absence of compensatory changes in the conventional T cell genes in snakes and lizards. Overall, the complexity of the γδ T cell subsets and the heterogeneity between species hinders our understanding of these immune cells.

## Building immune memory in γδ T cells

γδ T cells show features of both innate immunity, such as recognizing stress-related and pathogen-unspecific antigens, initiating ADCC and performing phagocytosis, and adaptive immunity, such as somatic rearrangement of receptor genes. Immune memory responses of γδ T cells have been documented in humans, cattle and mice (Table 1). With such unique characteristics that bridge innate immunity and adaptive immunity (Shen et al., 2002; Rincon-Orozco et al., 2005; Holtmeier and Kabelitz, 2005), a question arises: do γδ T cells mount adaptive immune memory, innate immune memory (trained immunity) or both?

**Table 1.** Immune memory responses of gamma delta (γδ) T cells. BCG: Bacille Calmette-Guerin; CMV: Cytomegalovirus; HSV: herpes simplex virus; IMQ: imiquimod; MMR: measles-mumps-rubella; MPV: Mpox virus.

*Species*	*T cell subset*	*Experimental setting*	*Location*	*Adaptive immune memory responses*		*Innate immune memory responses*		*Ref*
				*Inducing agent*	*Immune memory response characteristics*	*Inducing agent*	*Immune memory response characteristics*	
**Human and non-human primates**	Vδ2	In vitro	Peripheral blood	BCG	Enhanced proliferation upon *M. tuberculosis* stimulation			Hoft et al., 1998; Kabelitz et al., 1991
	Vδ2	In vivo	Peripheral blood	BCG	Enhanced proliferation and IFN-γ production upon restimulation			Kabelitz et al., 1991
	γδ	In vivo	Pulmonary and peripheral blood	BCG	Enhanced proliferation upon reinfection			Shen et al., 2002; Lai et al., 2003
	Vδ2	In vivo	Peripheral blood	*Listeria monocytogenes*	Enhanced proliferation and effector function upon reinfection			Ryan-Payseur et al., 2012
	γδ	In vivo	Peripheral blood	*Plasmodium falciparum*	Enhanced proliferation and IFN-γ production upon restimulation			Teirlinck et al., 2011
	Vδ1	In vivo	Peripheral blood	*Plasmodium falciparum*	Clonal expansion, recurrent parasite-exposure driven expansion and differentiation			von Borstel et al., 2021; Rutishauser et al., 2020
	Vδ2	In vivo	Peripheral blood	SARS-CoV-2 mRNA vaccine	Enhanced proliferation and IFN-γ production upon revaccination			Terzoli et al., 2024
	Vδ2	In vivo	Peripheral blood	MPV	Enhanced proliferation and IFN-γ production upon rechallenge			Shao et al., 2009
	Vδ1	In vivo	Peripheral blood	CMV	Rapid proliferation and infection resolution after reinfection			Pitard et al., 2008
	Vδ2	In vivo	Pulmonary compartment			*Listeria monocytogenes*	Enhanced IFN-γ and perforin production; lower pulmonary pathology and less weight loss upon *M. tuberculosis* infection	Shen et al., 2019
	γδ	In vitro	Peripheral blood			BCG	Enhanced TNF and IFN-γ production upon *C. albican* challenge; transcriptional rewiring	Suen et al., 2024
	Vδ2	In vitro	Peripheral blood			HSV	Enhanced lysing ability of infected cells upon PHA or mycobacteria stimulation	Bukowski et al., 1994
	γδ	In vitro	Peripheral blood			MMR	Enhanced TNF and IFN-γ production upon CD3 stimulation; transcriptional and metabolic rewiring	Röring et al., 2024
**Mouse**	Vγ4Vδ1	In vivo	Intestinal mucosa	*Listeria monocytogenes*	Enhanced proliferation and infection clearance upon rechallenge			Sheridan et al., 2013
	Vγ4	In vivo	Intestinal epithelium	*Listeria monocytogenes*	Enhanced IL-17 production and clustering with *L monocytogenes* replication foci upon secondary infection			Romagnoli et al., 2016
	Vγ6	In vivo	Peritoneum, draining mediastinal lymph nodes	*Staphylococcus aureus*	Enhanced IL-17 production and infection clearance after reinfection			Murphy et al., 2014
	Vγ6	In vivo	Kidney	*Staphylococcus aureus*	Reduced renal bacterial load upon reinfection			Bertram et al., 2023
	Vγ1	In vivo	Liver, lung, spleen	MCMV	Enhanced proliferation and survival rate upon rechallenge			Khairallah et al., 2015
	γδ	In vitro	Liver, spleen	*Plasmodium chabaudi*	Enhanced CD107a expression and IFN-γ production upon rechallenge; transcriptional reprogramming			Kumarasingha et al., 2020
	Vγ4Vδ4	In vivo	Skin			IMQ	Enhanced proliferation and IL-17 production upon IMQ rechallenge	Ramírez-Valle et al., 2015
	Vγ4Vδ4	In vivo	Skin; ear			IMQ	Enhanced proliferation and IL-17 production and neutrophil recruitment upon IMQ rechallenge	Hartwig et al., 2015
	Vγ4Vδ1	In vitro	Gut; bulk mesenteric lymph nodes			*Listeria monocytogenes*	Enhanced proliferation and IFN-γ and IL-17A production upon *S. enterica* serovar Typhimurium and *C. rodentium* challenge	Khairallah et al., 2022
**Cow**	γδ	In vitro	Airway and peripheral blood	BCG	Increased IFN-γ producing γδ T cells			Guerra-Maupome and McGill, 2019
	γδ	In vitro	Peripheral blood			BCG	Enhanced IL-6 and TNF production upon *Escherichia coli*, LPS and Pam3CSK4 stimulation; epigenetic rewiring	Samuel et al., 2024

### Adaptive immune memory of human and non-human primate γδ T cells

Immune memory responses of primate γδ T cells are best characterized in the Vδ2 T cell population. Already in the early 80s, Hoft et al. observed a drastic expansion of γδ T cells after the in vitro stimulation with *Mycobacterium tuberculosis* of peripheral blood mononuclear cells (PBMCs) from BCG-vaccinated individuals compared to the non-vaccinated donors or placebo recipients (Hoft et al., 1998). The highly reactive γδ T cells were mainly Vδ2 T cell subsets, most likely Vγ9Vδ2 T cell subset (Kabelitz et al., 1991), and their enhanced responsiveness to *M. tuberculosis* after BCG vaccination was shown to be independent of the helper function of CD4 T cells. Apart from greater expansion, the frequencies of IFN-γ-producing cells upon in vitro re-stimulation were also increased among γδ T cells from BCG-vaccinated individuals (Suliman et al., 2016). Similar observations were made in BCG-vaccinated macaques, which displayed an enhanced expansion of peripheral blood and pulmonary Vδ2 T cells upon reinfection with BCG or *M. tuberculosis* compared to unvaccinated animals (Shen et al., 2002; Lai et al., 2003). This coincided with improved microbial clearance, suggesting a protective role of the Vδ2 T cell recall responses, which were shown to be specific to the mycobacterial antigens. Altogether, these studies unraveled the potential of γδ T cells to mount adaptive immune memory responses specific to mycobacterial infection. Interestingly, neither BCG vaccination of infants nor re-vaccination of adults induced T cell memory phenotype in γδ T cells (Gela et al., 2022) defined by the expression of CD45RA and CCR7 surface molecules (Sallusto et al., 2004; Sallusto et al., 1999). Although frequencies of terminally differentiated effector CD45RA^+^CCR7^-^γδ T cells increased after BCG vaccination, this was accompanied by the increase in frequencies of naïve CD45RA^+^CCR7^+^ and a decrease in effector memory CD45RA^-^CCR7^-^γδ T cells (Gela et al., 2022). Pathogen-specific recall responses of Vδ2 T cells have also been reported in *L.monocytogenes* infection (Ryan-Payseur et al., 2012). Rhesus macaques systemically infected with an attenuated *L. monocytogenes* strain displayed a markedly enhanced in vivo proliferation and effector function, such as cytokine and cytotoxic molecule production and lysing potential of *L. monocytogenes*-infected cells, by peripheral blood Vδ2 T cells. This subset was also enriched in lung and intestinal mucosa after the secondary challenge with the pathogen compared to the initial challenge. The reduced pathogen burden in the circulation was associated with this increased responsiveness of Vδ2 T cells. The antigen involved in triggering the primary and recall immune responses of Vδ2 T cells, especially Vγ9Vδ2 T cells, in bacterial infections is most likely HMBPP, which is produced by both *Mycobacterium* (Bailey et al., 2002) and *Listeria* (Ryan-Payseur et al., 2012). It was used in in vitro stimulation assays of Vδ2 T cells from *L. monocytogenes-*exposed macaques to demonstrate the stronger induction of effector molecule-producing cells upon secondary infection (Ryan-Payseur et al., 2012). Yet, interestingly, in vitro exposure of human PBMCs to HMBPP and zoledronate resulted in a polyclonal proliferation of Vγ9Vδ2 T cells, questioning an adaptive character of the Vγ9Vδ2 T cell immune responses to phosphoantigens (Fichtner et al., 2020a; Papadopoulou et al., 2019).

Vδ2 T cells also respond to a live attenuated *P. falciparum* sporozoite (PfSPZ) vaccine (Ishizuka et al., 2016; Lyke et al., 2017). There is a strong indication that vaccine-induced Vδ2 T cells protect against *Plasmodium* infections since the expansion and frequency of γδ T cells in immunized volunteers was the best correlate of protection against controlled and naturally occurring malaria infections (Ishizuka et al., 2016; Lyke et al., 2017; Seder et al., 2013). Although the vaccine does not seem to affect the frequencies of Vδ1 T cell subset (Rutishauser et al., 2020), Vδ1 T cell proliferation had been observed in infected individuals from endemic malaria regions (Ho et al., 1994; Worku et al., 2003). Recently, a longitudinal analysis of blood samples collected throughout three malaria seasons in Mali showed that Vδ1 T cell frequencies increase after repeated exposure to seasonal episodes of febrile malaria and to controlled human malaria infection (CHMI) (von Borstel et al., 2021). This increase in Vδ1 T cell frequencies negatively correlated with the severity of symptoms. Interestingly, while the Vδ1 T cells from naïve individuals were unresponsive to trophozoite/schizont extracts (PfTSE), Vδ1 T cells exposed to *F. falciparum* in vitro or in vivo gained a proliferative response to the secondary exposure with PfTSE. Furthermore, the recurrent parasite exposure-driven expansion of Vδ1 T cells was accompanied by the differentiation of the cells towards cytotoxic effector phenotype defined as CD27^+^CX_3_CR1^+^, granzymes and perforin production as well as CD38 and CD16 expression. In contrast, the phenotype of Vγ9Vδ2 T cells upon serial reinfections remained predominantly unchanged. Similarly, the Vδ2^+^ T cell clonotypes remained stable throughout infections, while Vδ2^-^ T cells experienced dynamic changes to their TCR repertoire. Importantly, malaria infection or vaccination caused clonal expansion of Vδ2^-^ T cells, leading to overall focusing of Vδ1 TCR repertoire upon repeated exposure (von Borstel et al., 2021; Rutishauser et al., 2020). These results underpin the adaptive character of immune memory responses of Vδ1 T cells. Other CHMI trials have demonstrated that γδ T cells contribute to long-term immune memory responses against *P. falciparum* (Teirlinck et al., 2011). Similar to PfSPZ vaccination, the parasite activated and expanded peripheral blood γδ T cells (Teirlinck et al., 2011) and boosted the frequency of effector memory cells, defined as CD45RA^-^CD27^-^ (Mamedov et al., 2018). The number of *P. falciparum*-responsive IFN-γ-producing γδ T cells also increased after the secondary exposure of volunteers. Interestingly, although small in numbers, γδ T cells were the main producers of IFN-γ during the recall responses (Teirlinck et al., 2011). Yet, it remains unspecified in these studies which γδ T cell population exhibits enhanced functionality upon re-exposure to the pathogen. Altogether, this indicates that γδ T cells may mount specific immune memory responses upon parasitic infection and contribute to the longevity of malaria protection in humans.

Recently, the mRNA SARS-CoV-2 vaccine has been shown to induce a memory phenotype in Vδ2 T cells (Terzoli et al., 2024). While the first dose of vaccination had little effect on the transcriptome of Vδ1 and Vδ2 T cells, the vaccine booster caused profound changes. The revaccination induced central memory transcriptional signature and largely boosted the immune response of Vδ2 T cells, which was reflected by their increased expansion capacity and effector potential in vivo. This observation was further confirmed in vitro by exposing PBMCs from healthy donors to SARS-CoV-2 spike proteins. Upon re-exposure, Vδ2 T cells exhibited a higher IFN-γ production capacity compared to the primary stimulation, indicating an establishment of immune memory against the SARS-CoV-2 virus. Induction of transcription factors from the AP-1 family accompanied these functional changes. Vδ2 T cells have also been shown to respond to vaccinia virus immunization and Mpox virus (MPV) infection (Shao et al., 2009). In macaques, a suboptimal priming of Vδ2 T cells by vaccination with vaccinia virus administered together with the antiviral drug cidofovir led to an enhanced proliferative response of these cells upon subsequent MPV challenge compared to unvaccinated or vaccinated alone (without cidofovir) macaques (Shao et al., 2009).

Adaptive-like recall responses in response to viruses have also been described in Vδ1 T cells. Cytomegalovirus (CMV) seropositive subjects displayed a higher percentage of Vδ1 T cells with a more restricted TCR repertoire compared to CMV seronegative individuals (Pitard et al., 2008). Furthermore, Vδ1 T cells from CMV^-^ donors displayed a more naïve CD45RA^+^CD27^+^CD62L^+^ phenotype, while those in CMV^+^ individuals were predominantly of effector/memory CD45RA^+^CD27^-^CD28^-^CD62L^-^ phenotype (Pitard et al., 2008). Upon in vitro stimulation with CMV-infected fibroblasts, Vδ1 T cells from CMV^+^ patients strongly upregulated the CD107a marker, indicative of cytotoxic activity, compared to Vδ1 T cells from CMV^-^ individuals. Individuals who experienced CMV reactivation after immunosuppressive therapy displayed a rapid secondary expansion of Vδ2^-^ T cells and induction of effector/memory CD45RA^+^CD27^-^ phenotype, most likely of Vδ1 T cells, compared to individuals who only experienced primary infections. This faster response has been associated with a quicker resolution of the infection. Thus, it has been suggested that the CMV drives an expansion of memory-like Vδ1 T cells, which can readily respond to a CMV secondary challenge (Pitard et al., 2008). Altogether, the increased responsiveness of Vδ2 T cells after restimulation with a related pathogenic stimulus indicates the development of a pathogen-specific immune memory, similar to the classical adaptive immune memory of conventional T cells.

### Adaptive immune memory of murine γδ T cells

Immune memory responses have also been widely studied in different subsets of γδ T cells in mice. Intestinal and intrahepatic γδ T cells have been shown to not only provide immediate immunity by simultaneously producing IFN-γ and IL-17A (Hamada et al., 2008b) but also to develop immune memory upon *L. monocytogenes* infection (Sheridan et al., 2013; Romagnoli et al., 2016). Mice orally challenged with the pathogen experienced an induction of long-lived resident Vγ6 T cells in mesenteric lymph nodes and exhibited a more rapid and robust response upon secondary and tertiary oral challenge with *L. monocytogenes*, but it was not observed upon intravenous *Listeria* or oral *Salmonella* challenges. The memory γδ T cells protected the animals from secondary infection in a TCR-dependent manner, by clustering with myeloid cells at *L. monocytogenes* replication foci, producing IL-17A and cooperating with conventional T cells. Altogether, these observations indicate that murine Vγ6 T cells are also able to mount pathogen- and site-specific memory responses.

Similarly, γδ T cells also exert an immune memory response in *S. aureus* infection (Murphy et al., 2014; Bertram et al., 2023). γδ T cells, mostly Vγ6 subset, expanded vigorously upon intraperitoneal (Murphy et al., 2014) and intravenous infection with *S. aureus* (Bertram et al., 2023) and were retained in the draining mediastinal lymph nodes, peritoneum and kidney, respectively, for a prolonged period of time even after bacterial clearance. Upon reinfection, animals displayed enhanced expansion and IL-17 production by these cells and reduced bacterial load (Murphy et al., 2014). Furthermore, the adaptive transfer of *S. aureus*-experienced γδ T cells improved the outcome of subsequent infection (Murphy et al., 2014) and γδ T cell deficiency abrogated this protective effect (Bertram et al., 2023). Altogether, immune memory responses of Vγ6 T cells protect against *S. aureus* reinfection.

Immune memory responses against viral infection have also been observed in murine γδ T cells (Khairallah et al., 2015). In the absence of conventional T cells and NK cells, γδ T cells are sufficient to control murine CMV (MCMV) infection by restricting viral load in the liver, lung, and spleen. Numbers of γδ T cells, mostly Vγ1 but also Vγ4 subsets, surged in these organs after infection with the virus and displayed mainly effector memory (EM) phenotype defined as CD62L^-^CD44^+^. Adoptive transfer experiment to CD3ε knockout MCMV^-^ recipients showed that mice that received γδ T cells from MCMV^+^ donors had a higher survival rate upon MCMV infection than those that received γδ T cells from MCMV^-^ donors. Although the killing mechanism and effector response remain undefined, this observation suggests that γδ T cells may play an indispensable role against MCMV re-infections by exerting immune memory responses.

Similar to human γδ T cells, murine γδ T cells also imprint memory against parasitic infection after the initial exposure (Kumarasingha et al., 2020). *Plasmodium* infection causes activation and expansion of γδ T cells (Mamedov et al., 2018; Kumarasingha et al., 2020). Splenocytes extracted from *Plasmodium chabaudi*-infected mice presented a higher number of CD107a^+^ and IFN-γ-producing γδ T cells after in vitro challenge with *P. chabaudi*-infected red blood cells when compared to splenocytes from naïve mice (Kumarasingha et al., 2020). The responding cells were predominantly EM CD62L^-^CD44^+^ cells, although the frequencies of naïve, memory, and effector subsets did not vary between infected and naive animals. Consistently, γδ EM T cells displayed a similar transcriptional signature to CD8^+^ memory T cells, and genes related to cytokines, chemokines, antigen-presenting, and cytotoxic properties were upregulated in the γδ EM T cells of the previously infected mice, indicating a transcriptional rewiring, one of the characteristics of immune memory induction. Taken together, these observations suggest that murine γδ T cells are able to form pathogen-specific immune memory.

### Innate immune memory of human and non-human primate γδ T cells

As present reports focused on determining immune recall responses of γδ T cells in a pathogen-specific context, a recent study demonstrated the induction of a pathogen-unspecific memory response by γδ T cells (Shen et al., 2019). Macaques vaccinated with a bacterial-based vaccine containing an attenuated strain of *L. monocytogenes* exhibited a prolonged expansion of Vδ2 T cells in the circulation and pulmonary compartments, dependent on HMBPP production by the pathogen. After the infection of *L. monocytogenes-*immunized animals with *M. tuberculosis,* a higher number of IFN-γ- and perforin-producing Vδ2 T cells in the airway of the immunized animals was observed compared to the HMBPP-deficient *L. monocytogenes* strain, which contributed to the inhibition of the intracellular *M. tuberculosis* growth. As a result, the immunized macaques presented a lower pulmonary pathology and less weight loss upon the infection with unrelated bacteria (Shen et al., 2019). This study demonstrated for the first time the development of pathogen-unspecific but HMBPP-dependent memory responses by primate γδ T cells.

Further studies illustrated the potential of innate immune memory development by human γδ T cells (Suen et al., 2024). Individuals who were vaccinated with BCG, a well-known trained immunity inducing agent (Kleinnijenhuis et al., 2012; Arts et al., 2016; Quintin et al., 2014; Li et al., 2023a), presented a higher percentage of perforin-producing γδ T cells in the blood and higher numbers of IFN-γ-producing γδ T cells upon in vitro stimulation with *M. tuberculosis* compared to the non-vaccinated controls, consistent with previously shown establishment of adaptive immune memory in γδ T cells upon mycobacteria rechallenge. Importantly, the in vitro stimulation of PBMCs from BCG-vaccinated individuals with BCG-unrelated heat-inactivated *C. albicans* also caused an increased fold change of TNF- and IFN-γ producers within γδ T cells before and after vaccination vs. unvaccinated individuals, suggesting the development of a trained immunity phenotype. Moreover, the γδ T cells of approximately half of the BCG-vaccinated individuals displayed an enhanced cytokine production capacity upon PBMC stimulation with heat-inactivated *E. coli* and *S. aureus* when compared to non-vaccinated individuals. This observation is consistent with the previous studies showing that there is a high interindividual heterogeneity in the capacity to mount trained immunity in response to BCG (Moorlag et al., 2024). Single-cell RNA sequencing data further demonstrated that *IFNG* expression was upregulated in a specific subset of γδ T cells in the PBMCs of vaccinated individuals when rechallenged by lipopolysaccharide (LPS) 3 months after the vaccination. This indicated a trained immunity induction in γδ T cells at the transcriptional level. Interestingly, cell-cell communication analysis suggested the role of *IFNG*-related communication between human γδ T cells and monocytes for the formation of the innate immune response. Here, the study confirmed the induction of innate immune memory in γδ T cells upon bacterial challenges.

Although the activation mechanisms of Vδ2 T cells in viral infections are not fully understood, innate immune memory responses of Vδ2 T cells upon viral challenges have been reported for herpes simplex virus (HSV) (Bukowski et al., 1994). For example, stimulation of PBMCs from HSV seropositive patients with autologous HSV-infected Phytohemagglutinin (PHA) blasts induced an expansion of Vδ2 T cells HSV (Bukowski et al., 1994). Surprisingly, the Vδ2 T cells previously simulated by PHA or mycobacteria showed an increased lysing ability of HSV-infected cells compared to the mock target. This enhanced lysing ability of Vδ2 T cells has also been observed against vaccinia-infected cells, the infectious agent not related to HSV (Bukowski et al., 1994). The authors suggested that the Vδ2 T cell effector function depends on TCR activation, where the TCR ligands are not specific viral antigens but rather originate from the modulation of cellular components of infected cells by the virus.

MMR vaccine is another vaccine besides BCG known to contribute to heterologous protection against nontarget infections (Sørup et al., 2014; Tielemans et al., 2017). Single-cell RNA sequencing (scRNAseq) of the PBMCs isolated from MMR-vaccinated volunteers revealed that γδ T cell population experiences the biggest transcriptional changes among the cellular components of PBMCs after vaccination (Röring et al., 2024). Furthermore, the transcriptome analysis and energy metabolic profiling unraveled a higher protein synthesis and an alteration of glycolytic capacity and mitochondrial dependency of Vδ2 T cells of vaccinated individuals upon restimulation when compared to the non-vaccinated individuals. Furthermore, the TNF and IFN-γ production by Vδ2 T cells significantly increased in MMR-re-vaccinated volunteers when compared to the non-vaccinated donors. Such transcriptional and metabolic rewiring and enhanced responsiveness of Vδ2 T cells against secondary stimulation resemble the classical phenotype of trained immunity in monocytes.

### Innate immune memory of mouse γδ T cells

Evidence of trained immunity induction has also been documented in murine γδ T cells. Two similar studies have revealed that local skin inflammation established long-lived memory Vγ4^+^Vδ4^+^ T cells capable of enhanced inflammatory reaction upon rechallenge with innate-like ligand imiquimod (IMQ) (Ramírez-Valle et al., 2015; Hartwig et al., 2015). After the initial application of the IMQ, the IL-17-producing γδ T cell population, mainly Vγ4^+^Vδ4^+^ T cells, populates the dermis and migrates via blood to lymph nodes and distal skin sites, where it persists for months. Upon re-challenge with IMQ, memory-like Vγ4^+^Vδ4^+^ T cells displayed a rapid secondary expansion and produced a higher amount of IL-17 than upon primary challenge, leading to an escalated skin inflammatory response (Ramírez-Valle et al., 2015). Furthermore, the inflamed sites were associated with heightened neutrophil recruitment and more rapid ear thickening upon re-challenge, which was dependent on Vγ4^+^Vδ4^+^ T cell presence, indicating that γδ T cell immune memory responses orchestrate other cells of the immune system (Hartwig et al., 2015). Transfer experiments confirmed that memory-like Vγ4^+^Vδ4^+^ T cells exhibit enhanced responsiveness (Ramírez-Valle et al., 2015). The memory Vγ4^+^Vδ4^+^ T cells displayed increased IL-1R1 surface expression and proliferation in response to IL-1β, suggesting that intrinsic increased sensitivity to IL-1β signaling is the acquired adaptation of memory γδ T cells that allows them to respond more rapidly to a secondary challenge (Ramírez-Valle et al., 2015). Such a rapid and robust secondary response to the innate ligands indicates a potential induction of trained immunity in murine γδ T cells, yet there is a certain specificity as the response to mannan, a polysaccharide causing skin inflammation by activating mannose receptors (Wu et al., 2023), was not enhanced in the IMQ-sensitized mice (Ramírez-Valle et al., 2015). Interestingly, the recall responses seem to be mediated by TCR activation even though IMQ is a TLR –7/8 ligand, suggesting the engagement of numerous immune receptors on γδ T cells (Hartwig et al., 2015). Interestingly, intestinal memory Vγ4^+^Vδ1^+^ T cells generated in response to food-borne *L. monocytogenes* infection seem to be reactive to unrelated bacteria such as *Salmonella enterica* serovar Typhimurium and *Citrobacter rodentium* when rechallenged ex vivo and to *Yersinia pseudotuberculosis* when rechallenged in vivo (Khairallah et al., 2022). Thus, these findings indicate the broadly reactive nature of memory Vγ4^+^Vδ1^+^ T cells, suggesting the potential to induce a trained immunity phenotype in these cells.

### Immune memory of bovine γδ T cells

Immune memory responses of γδ T cells have also been reported in other mammals, especially in calves (Guerra-Maupome et al., 2019). *M. bovis* infection triggered proliferation of CD27^+^ γδ T cell memory subset in the circulation (Guerra-Maupome et al., 2019). The IFN-γ-producing γδ T cells in circulation and in the airway of BCG-vaccinated cows significantly increased upon stimulation by purified protein derivatives from *M. bovis* 8 weeks after the vaccination (Guerra-Maupome and McGill, 2019). These studies indicate the induction of adaptive immune memory responses in bovine γδ T cells. Besides, a recent study showed that γδ T cells from BCG-vaccinated calves present an increased IL-6 and TNF production upon stimulation with *E. coli*, LPS and Pam3CSK4 (Samuel et al., 2024). Importantly, chromatin accessibility analysis further revealed increased promoter accessibility of certain innate immunity-related genes in the trained bovine γδ T cells, which is consistent with the common phenotype of epigenetic reprogramming in trained immunity. Altogether, these observations suggest that BCG can elicit adaptive and innate immune memory responses in bovine γδ T cells.

## Perspectives

The capacity of γδ T cells to mount immune memory responses against the same and unrelated pathogens has been reported, yet the precise molecular basis of this immune memory formation remains to be unveiled. This is an emerging research area and although distinct memory features of γδ T cells have been demonstrated, several questions remain to be addressed to advance the current understanding of the adaptive and/or innate memory of γδ T cells. Key questions related to immune memory of γδ T cells awaiting to be answered are: (1) What are the receptors on γδ T cells that are engaged during the initial and secondary exposures? (2) What is the role of metabolism in the immune memory formation by γδ T cells? (3) Which epigenetic mechanisms are involved? (4) To what extent are processes conserved between species and between different γδ T cell subsets? And finally, can the trained immunity potential of γδ T cells be harnessed for therapeutic interventions? Do trained γδ T cells alter host defense by regulating endocrine circuits (Šestan et al., 2024) with an impact on antimicrobial defense? Whether adaptive and innate memory are counter or co-regulated in γδ T cells remains elusive as well. The current state of knowledge is still insufficient to unambiguously answer the question whether γδ T cells are capable of exerting both innate and adaptive immune memory responses. This question is difficult to answer primarily due to the fact that ligands recognized by γδ TCRs are largely unknown. Another challenge stems from the fact that different γδ T cell populations display distinct features, for example, Vδ1 and Vγ9^-^Vδ2^+^ being of adaptive while Vγ9^+^Vδ2^+^ being of innate character (Davey et al., 2018; von Borstel et al., 2021; Davey et al., 2017; Ravens et al., 2017; Rutishauser et al., 2020), or that γδTCRs have a dual reactivity, such as human and mouse intestinal γδTCRs use spatially distal regions to recognize non-clonal agonist-selecting elements by germline-encoded segments, and clone-specific ligands by the complementary-determining regions (CDRs) (Melandri et al., 2018). The current advancements in the next generation sequencing technologies allowing to address clonal expansion of cells carrying γδTCRs will help to solve this mystery in the near future. We anticipate that detailed knowledge about trained immunity will enable the development of γδ T cell-targeted approaches to trigger or restrict this memory feature for the benefit of the host. γδ T cell-focused host-directed interventions could help prevent and cure infections, but only when the answers to these questions are known will we be able to fully exploit the therapeutic potential of γδ T cells.
